# Dietary macroalgae *Chaetomorpha linum* supplementation improves morphology of small intestine and pectoral muscle, growth performance, and meat quality of broilers

**DOI:** 10.14202/vetworld.2024.470-479

**Published:** 2024-02-23

**Authors:** H. T. Saragih, I. N. Fauziah, D. A. Saputri, A. R. Chasani

**Affiliations:** 1 Laboratory of Animal Development Structure, Faculty of Biology, Universitas Gadjah Mada, Sleman, Yogyakarta 55281, Indonesia; 2 Graduate Program of Biology, Department of Tropical Biology, Universitas Gadjah Mada, Sleman, Yogyakarta 55281, Indonesia; 3 Laboratory of Plant Systematics, Faculty of Biology, Universitas Gadjah Mada, Sleman, Yogyakarta 55281, Indonesia

**Keywords:** *Chaetomorpha linum*, growth performance, pectoral muscle, small intestine, water-holding capacity

## Abstract

**Background and Aim:**

Over the last decades, the poultry industry has experienced steady growth. Although the industry is gradually expanding in Indonesia, poultry feed production has always been expensive. There is a need to study alternative ingredients to obtain affordable feed from natural resources. Chaetomorpha linum (CL) is an abundant macroalgae available throughout the year in Indonesia. This study aimed to determine the effect of CL on the histological structure of the small intestine, pectoralis muscle, growth performance, and meat quality of broilers.

**Materials and Methods:**

This study used 300-day-old chick (DOC) male broilers that were reared until they were 21 days old. This study used a completely randomized design with four treatment groups and five replications, and each replication group contained 15 DOC individuals. The treatment groups consisted of Control (CON), CON basal feed (BF), CL1 (0.75%/kg BF), CL2 (1.5%/kg BF), and CL3 (3%/kg BF) groups. The histological structure of the small intestine, pectoralis muscle, growth performance, and meat quality of the broiler was examined.

**Results:**

Small intestine and pectoral muscle histomorphology, growth performance, and meat quality were significantly improved in the CL2 (1.5%) and CL3 (3%) groups compared with the CL1 (0.75%) and CON groups.

**Conclusion:**

Dietary CL supplementation ameliorates small intestine and pectoral muscle histomorphology, growth performance, and meat quality of broilers.

## Introduction

As an important agricultural sector, Indonesia’s poultry industry has experienced a steady growth in recent decades [1]. Although the poultry industry has gradually expanded, animal feed production is constantly expensive in Indonesia. This is mainly due to the lack of availability of the ingredients. Animal feed ingredients, such as soybean meal, corn, vitamins, and supplements, are not entirely locally accessible since most of them are still imported [2, 3]. Minimizing feed costs is a major concern in the poultry industry [4]. Therefore, some alternatives derived from natural ingredients have been studied to meet the need for affordable animal feed [5]. Marine products such as macroalgae have a long history of being incorporated into animal feed [6, 7]. Conventionally, macroalgae have a high potential to be used as chicken feed. This is due to the abundance of macroalgae in nature [8]. Ecologically, if it is not utilized, it will exacerbate environmental disturbances, such as reducing the intensity of light entering the seawater, depleting dissolved oxygen levels, and blooming into invasive species that grow in seagrass beds, thus endangering seawater organisms [9–11]. Furthermore, macroalgae have diverse nutritional profiles, including carbohydrates, protein, lipids, minerals, dietary fiber, balanced amino-acid content, and bioactive compounds, which make them a potential candidate for inclusion in chicken diets [7, 12].

Several studies on the inclusion of macroalgae in chicken diet have shown promising results. A study utilizing red macroalgae *Kappaphycus alvarezii* in broiler feed resulted in improved meat quality [13]. Another study showed that supplementation with the green macroalgae *Ulva rigida* extensively increased the villi length of the intestine and led to better absorption of nutrition in broilers [14]. Hafsa and Hassan [15] also reported the effect of enhancing intestinal villi length and width after supplementing *Sargassum siliquastrum* into a quail diet. A combination of *Undaria pinnatifida* and *Hizkia fusiformis* has also been shown to improve overall body weight gain and egg-laying performance in laying hens [16]. Considering that Indonesia is one of the world’s major macroalgae producers with more than 500 macroalga species [17], the prospect of utilizing macroalgae as animal feed is promising. A locally available and easily accessible natural ingredient may be a good alternative to animal feed. The abundance and diversity of marine macroalgae may lead to promising findings because the global interest in marine macroalgae as a sustainable feedstock for feed ingredient production is increasing [12]. The use of marine macroalgae as animal feed, particularly poultry feed, is a sustainable way of conserving and strengthening the poultry industry at the same time. *Chaetomorpha linum* (CL) is an abundant species along the coastal line in Indonesia [18]. CL is a bloom-forming algae that can be found around the globe [10]. This species is characterized by filamentous, unbranched, and stiff thallus with bright green to dark green color [19], living in colonies. Gayathiri *et al*. [20] reported that CL is an important source of natural antioxidants with abundant secondary metabolic activity. CL polysaccharides also have promising potential as antidiabetic agents [21].

Several studies of CL as animal feed have been conducted on shrimp and fish as well as on other livestock, particularly ruminants [22–24]. The growth performance of the white shrimp *Litopenaeus vannamei* increased following treatment with CL macroalgae [23]. A combination of *Ruppia* *maritima* and CL can be used as an alternative feed resource for lamb fattening during drought [24]. Song *et al*. [25] demonstrated that feeding CL to sea cucumber *Apostichopus japonicus* resulted in better growth and absorption of nutrition. Another study reported that CL polysaccharides improved heart function and modulated lipid metabolism in hyperlipidemic mice [26].

Although the application and research of CL as an animal feed and bioactive compound resource has been increasing, its use in the chicken industry has not yet been widely investigated. Therefore, this study aimed to investigate the histological structure of the small intestine and pectoral muscle, meat quality, and growth performance of broilers following CL supplementation.

## Materials and Methods

### Ethical approval

The Institutional Animal Care and Use Committee Veterinary Faculty of Gadjah Mada University approved (002/EC-FKH/Eks./2023) all animal handling, sampling, care, and experiment procedures on January 9, 2023.

### Study period and location

This study was conducted from June to September 2023 at the Sawitsari Research Station and Laboratory of Developmental Structures and Animal Histology, Faculty of Biology, Gadjah Mada University.

### Diet formulation

Basal feed (BF) was acquired from a local supplier by PT Sari Rosa Asih, Indonesia. The nutrient and proximate composition of BF is presented in Table-1 [27]. CL was collected from Sepanjang Beach, Yogyakarta, Indonesia and identified at the Plant Systematics Laboratory, Faculty of Biology, Gadjah Mada University. The collected CL was sun-dried and incubated for 24 h before being ground and crushed into powder.

**Table-1:** Basal feed formulation and nutrient content of broiler chicken for 21 days.

Composition of feed (%)	Single feed
Corn	49
Soybean meal	29
Rice bran	9.8
Full-fat soya	5.4
Crude palm oil	3
Dicalcium phosphate	2.37
Premix vitamin^a^	0.03
Premix mineral^b^	0.06
D, L-methionine	0.22
NaCl	0.32
Calcit	0.5
L-lysine HCl	0.1
L-threonine	0.04
Choline chloride 60%	0.16
Calculated composition^c^	
Metabolizable energy of poultry (kcal/kg)	2,904.02
Crude protein (%)	20.23
Crude fat (%)	8.3
Fiber (%)	3.37
Lysine (%)	1.22
Methionine (%)	0.53
Methionine + cysteine (%)	0.86
Calcium (%)	1
Phosphorus, total (%)	0.95
Phosphorus, available (%)	0.5
Sodium (%)	0.15
Chloride (%)	0.23

a Premix vitamin provided the following per kilogram of diet (Vitamin A: 15000 IU, Vitamin D3: 3000 IU, Vitamin E: 22.5 mg, Vitamin K3: 3 mg, Vitamin B1: 3 mg, Vitamin B2: 9 mg, Vitamin B6: 4.5 mg, Vitamin B12: 30 mcg, biotin: 30 mcg, folic acid: 1.5 mg, niacin: 45 mg, pantothenic acid: 1.5 mg, Vitamin C: 0 mg, choline: 2090 mg and 1242 mg),

b Premix mineral provided the following per kilogram of diet (Cu: 12 mg, Fe: 72 mg, Iodine: 0.9 mg, Mn: 84 mg, Se: 0.3 mg, Zn: 60 mg),

c Proximate, amino acids, minerals, and metabolizable energy were obtained from calculated values

### Birds, husbandry, and experimental design

A total of 300 1-day-old male broilers (Cobb 500) (47–48 g) were obtained from a local hatchery by PT Japfa Comfeed, Indonesia, and randomly assigned into four treatment groups: (1) BF diet control (CON), (2) BF supplemented with 0. 75% CL (CL1), (3) BF supplemented with 1.5% CL (CL2), and (4) BF supplemented with 3% CL (CL3). The birds were housed in 85 × 60 cm pens equipped with artificial lighting (24 light photoperiods), 35°C–36°C temperature, *and water and feed ad libitum* for 5 replications and 15 birds per replication. After the acclimation period, the birds were fed on day 4 post-hatch and randomly euthanized on day 21 using the neck decapitation method with 1 bird per replication.

### Calorie, proximate, and flavonoid test results of CL

The proximate CL calorie and flavonoid tests were performed at the Center of Food and Nutrition Laboratory, Gadjah Mada University, Indonesia. The proximate and calorie tests evaluated the water, ash, fat, protein, carbohydrate, and calorie contents in CL in percent units. Flavonoid analysis was performed using a Microlab 300 Ultraviolet (UV)–vis spectrophotometer (Thermo Fisher Genesys 10s, Massachussets, USA) to analyze flavonoid content.

### Growth performance

Growth performance was measured using body weight, feed intake, and feed conversion ratio (FCR). Body weight was measured at 0, 4, 7, 14, and 21 days after hatching. The amount of feed given was calculated every day for 21 days. FCR was calculated using the total feed per kilogram consumed divided by body weight gain to determine the ratio of the amount of chicken feed needed with the increase in body weight during the treatment.

### Sample preparation

Broilers aged 21 days were fasted for 6 h before slaughter. Dissection was performed on the ventral side using scissors and a scalpel. The small intestine and pectoralis muscle were collected for sampling. The duodenum, jejunum, and ileum were taken from the small intestines, and the pectoralis major muscle sample was taken. The intestine was irrigated using physiological saline (NaCl 0.9%), followed by the pectoral muscle.

### Histological analysis of the small intestine and pectoral muscle

The small intestine was histologically prepared by separating the sections between the duodenum, jejunum, and ileum. A transverse section of the organ was used to observe the villi length, crypt depth, and number and area of goblet cells. The intestine was then stained with Periodic Acid Schiff-Alcian Blue (PAS-AB). This stain is used to detect PAS and AB in the intestinal mucosa. The right pectoralis major muscle was taken and cut to a size of 1 × 1 cm. The pectoralis major muscle was then stained with hematoxylin-eosin [28]. The villi, crypts, and goblet cells of the small intestine, as well as the fasciculus and myofiber of the pectoralis major, were observed and documented using a Leica DM750 microscope (Leica Microsystems, Wetzlar, Germany) at 4× and 10× magnification. For each preparation, image capture was performed using nine fields of view. Villi length, crypt depth, goblet cell area, fasciculus, and myofiber area were measured using ImageJ software (National Institutes of Health and the Laboratory for Optical and Computational Instrumentation, LOCI, University of Wisconsin, USA) [29].

### Meat quality and cholesterol test results

Meat quality was measured by cutting a 1 × 1 cm sample of breast muscle. Meat quality testing was conducted at Gadjah Mada University Faculty of Animal Husbandry. Water holding capacity (WHC) was the indicator tested in this study in each treatment group. WHC measurements were performed using the Hamm method as described by Abdullah *et al*. [30]. A cholesterol test was carried out using a Microlab 300 UV–vis spectrophotometer (Thermo Fisher Genesys 10s).

### Statistical analysis

The data obtained were tested for distribution patterns and homogeneity tests, followed by a one-way analysis of variance for data that followed a normal and homogeneous distribution pattern. The results of this analysis were then carried out with a follow-up test using Duncan’s test to show differences between the treatment groups. Test results are considered statistically significant at p ≤ 0.05. All data were analyzed using Statistical Package for the Social Sciences (SPSS) 25.0 software for Windows (IBM SPSS, Inc., NY, USA).

## Results

### Proximate, calorie, and flavonoid content

Table-2 shows the results of the proximate and calorie tests for CL. The water content was 3.47%, ash 40.85%, fat 2.25%, protein 15.48%, carbohydrates 73.43%, and calories 2234 cal/g. The flavonoid test results are shown in Table-3 with a flavonoid concentration of 0.16% w/w.

**Table-2:** Nutrient content of *Chaetomorpha linum*.

Content	Result	Unit
Water	3.47	%
Ash	40.85	%
Fat	2.25	%
Protein	15.48	%
Carbohydrate	37.43	%
Calorie	2,234	Kcal ME/kg

**Table-3:** Flavonoid content of *Chaetomorpha linum*.

Parameter	Result (w/w)
Flavonoid Total	0.16%

### Broiler growth performance

The effects of CL supplementation on chicken growth performance are summarized in Table-4. An increase in body weight gain occurred in all treatment groups. Significant differences in body weight gain occurred from day 7 to day 21 in all treatment groups compared to the CON group. Broiler feed intake was significantly different between the CON and treatment groups. Because body weight in the treatment group increased over a wide range, it affected the FCR value. As shown in Table-4, the FCR values of the CON and the treatment groups were significantly different.

**Table-4:** Broiler chicken growth performance after CL supplementation at 21 days old.

Variables	Day	Treatments				p-value

		CON	CL1	CL2	CL3	
Body Weight (g)	1	48.2 ± 1.462	47.4 ± 2.227	47.48 ± 1.480	48.96 ± 2.036	0.517
	7	111.64 ± 4.786^d^	113.16 ± 2.539^c^	121.76 ± 5.824^b^	128.12 ± 5.432^a^	0.000
	14	299.44 ± 3.559^d^	319.08 ± 3.087^c^	341.68 ± 7.694^b^	359.32 ± 7.585^a^	0.000
	21	547.8 ± 10.521^d^	566.8 ± 10.183^c^	633.4 ± 7.266^b^	752.2 ± 16.146^a^	0.000
Weight Gain (g/day ages)		23.89 ± 0.207^d^	24.90 ± 0.15^c^	27.92 ± 0.60^b^	32.93 ± 0.27^a^	0.000
Feed Intake (g/day ages)		38.755 ± 0.846^a^	37.691 ± 0.626^a^	26.420 ± 0.98^c^	29.514 ± 0.187^b^	0.000
Feed Convention Ratio (g/day ages)		1.66 ± 0.167^a^	1.60 ± 0.287^a^	1.31 ± 0.208^b^	1.29 ± 0.164^b^	0.023

CON=Control, BF=Basal feed, CL=*Chaetomorpha linum*, CL1: 0.75% *Chaetomorpha linum* supplemented/kg BF, CL2: 1.5% *Chaetomorpha linum* supplemented/kg Bf, CL3: 3% *Chaetomorpha linum* supplemented/kg BF. (Mean ± Standard error of mean). ^a-d^Differences in notation behind numbers in the same row indicate significant differences in values (p < 0.05)

### Histological structure of the small intestine

The histological structure of the small intestine of broilers supplemented with CL is presented in Table-5 as the mean ± Standard error of mean (SEM). The villi lengths in the duodenum, jejunum, and distal ileum were significantly longer in the CL treatment group (p < 0.05) than in the CON treatment group. A similar thing happened with regard to the depth of the crypts of the duodenum, jejunum, and ileum. The CL treatment groups showed the lowest significant value (p ≤ 0.05) compared to the CON group. The ratios of villi/crypts in the duodenum, jejunum, and distal ileum were also significantly different between the CL and CON treatment groups.

**Table-5:** Histological structure of small intestine in broiler chickens after giving CL treatment after 21 days old.

Variable	Treatments				p-value

	CON	CL1	CL2	CL3	
Duodenum					
Villi length (μm)	749.349 ± 16.968^c^	855.832 ± 14.217^b^	892.876 ± 19.572^b^	1090.200 ± 6.482^a^	0.000
Crypt depth (μm)	116.025 ± 5.807^d^	211.557 ± 3.221^c^	254.577 ± 3.305^b^	302.557 ± 5.192^a^	0.000
Villi/Crypt Ratio	6.538 ± 0.419^a^	4.051 ± 0.110^b^	3.505 ± 0.036^b^	3.606 ± 0.043^b^	0.000
Goblet Cell Area (μm^2^)	40.103 ± 0.910^d^	52.564 ± 0.395^c^	60.308 ± 1.131^b^	92.206 ± 1.232^a^	0.000
Number of Goblet Cells	140.644 ± 0.922s	223.399 ± 2.437^c^	254.022 ± 4.154^b^	408.777 ± 4.520^a^	0.000
Jejunum					
Villi length (μm)	513.371 ± 4.126^d^	611.588 ± 4.343^c^	650.057 ± 3.556^b^	803.082 ± 13.174^a^	0.000
Crypt depth (μm)	86.234 ± 1.238^c^	93.088 ± 1.042^b^	95.171 ± 0.276^b^	113.004 ± 0.528^a^	0.000
Villi/crypt ratio	5.959 ± 0.116^c^	6.572 ± 0. 076^bc^	6.830 ± 0.042^b^	7.108 ± 0. 135^a^	0.000
Goblet cell area (μm^2^)	39.603 ± 0.835^d^	52.025 ± 3.244^c^	66.385 ± 1.441^b^	78.721 ± 1.432^a^	0.000
Number of goblet cells	93.333 ± 1.133^d^	110.400 ± 3.692^c^	130.577 ± 1.478^b^	237.888 ± 1.081^a^	0.000
Distal ileum					
Villi length (μm)	410.551 ± 1.853^d^	551.910 ± 2.558^c^	581.557 ± 6.638^b^	702.988 ± 6.082^a^	0.000
Crypt depth (μm)	60.486 ± 2.196^d^	71.285 ± 1.233^c^	77.434 ± 0.751^b^	91.280 ± 1.179^a^	0.000
Villi/crypt ratio	6.819 ± 0. 219^b^	7.749 ± 0. 104^a^	7.512 ± 0.100^b^	7.703 ± 0.056^b^	0.001
Goblet cell area (μm^2^)	36.757 ± 2.470^d^	52.189 ± 1.139^c^	60.722 ± 0.849^b^	65.702 ± 1.019^a^	0.000
Number of goblet cells	70.555 ± 1.245^d^	80.311 ± 1.256^c^	85.844 ± 1.008^b^	110.311 ± 1.635^a^	0.000

CON=Control, Bf=Basal feed, CL=*Chaetomorpha linum*, CL1: 0.75% *Chaetomorpha linum* supplemented/kg Bf, CL2: 1.5% *Chaetomorpha linum* supplemented/kg BF, CL3: 3% *Chaetomorpha linum* supplemented/kg BF. (Mean ± Standard error of mean). ^a-d^Differences in notation behind numbers in the same row indicate significant differences in values (p < 0.05)

The area of goblet cells in the duodenum, jejunum, and distal ileum was significantly greater in the CL treatment group (p ≤ 0.05) than in the CON treatment group. The CL treatment groups observed variable numbers of goblet cells in the duodenum, jejunum, and distal ileum (p < 0.05) compared to the CON group.

### Pectoralis major

Table-6 shows the effect of the incorporation of CL supplements on the pectoral muscle growth of broiler chickens. All treatment groups had higher values than the CON group. The data shown in the table are presented as mean ± SEM. The weight of the pectoral muscles in the CL treatment group was significantly higher than that in the CON group. In addition, there was a significantly greater difference in the pectoral muscle area in the CL treatment groups than in the CON group. The fasciculus areas were significantly larger in all treatment groups than in the CON group. However, in terms of area of myofibers, each group showed a significant difference compared to the CON. A significant difference in the total number of myofibers was also observed between the treatment and CON groups.

**Table-6:** Pectoral muscle histoarchitecture of broiler chicken after CL supplementation at 21 days old.

Variable	Treatments				p-value

	CON	CL1	CL2	CL3	
Muscle Weight (g)	30.13 ± 3. 010^c^	43.76 ± 2.278^bc^	49.11 ± 0.85^b^	56.27 ± 3.14^a^	0.000
Muscle Area (cm)	45.60 ± 1.39^d^	51.44 ± 0.98^c^	61.49 ± 0.38^b^	64.63 ± 0.57^a^	0.000
Fascicle Area (mm^2^)	76374.27 ± 1839.41^d^	162331.58 ± 2685.87^c^	179406.22 ± 1265.51^b^	253959.76 ± 3017.21^a^	0.000
Myofiber Area (mm^2^)	769.59 ± 26.39^c^	897.86 ± 35.95^b^	990.93 ± 31.96^b^	1339.22 ± 49.26^a^	0.000
Total Myofiber	146.66 ± 5.48^d^	164.06 ± 2.68^c^	204.70 ± 3.89^b^	243.90 ± 7.17^a^	0.000

CON=Control, Bf=Basal feed, CL=*Chaetomorpha linum*, CL1: 0.75% *Chaetomorpha linum* supplemented/kg Bf, CL2: 1.5% *Chaetomorpha linum* supplemented/kg BF, CL3: 3% *Chaetomorpha linum* supplemented/kg BF. (Mean ± Standard error of mean). ^a-d^Differences in notation behind numbers in the same row indicate significant differences in values (p < 0.05)

### Meat Quality (WHC)

Table-7 shows the effect of CL on the meat quality of broilers. An indicator used to determine meat quality is the water-holding capacity of the meat sample. The CL2 and CL3 groups had significantly higher values compared to the CL1 and CON groups.

**Table-7:** Water holding capacity of broiler meat after CL supplementation for 21 days.

Variables	Treatments				p-value

	CON	CL1	CL2	CL3	
Water holding capacity (%)	31.702 ± 3.288^b^	28.000 ± 3.860^b^	37.088 ± 2.442^a^	38.316 ± 2.276^a^	0.000

CON=Control, BF=Basal feed, CL=*Chaetomorpha linum*, CL1: 0.75% *Chaetomorpha linum* supplemented/kg BF, CL2: 1.5% *Chaetomorpha linum* supplemented/kg BF, CL3: 3% *Chaetomorpha linum* supplemented/kg BF. (Mean ± Standard error of mean). ^a-b^Differences in notation behind numbers in the same row indicate significant differences in values (p < 0.05)

### Cholesterol

Table-8 shows the cholesterol test results for broiler meat samples after CL supplementation. There was no significant difference between the CON and treatment groups.

**Table-8:** Cholesterol concentration of broiler meat after CL supplementation for 21 days.

Variables	Treatments				p-value

	CON	CL1	CL2	CL3	
Cholesterol (mg/mL)	45.382 ± 1.852	45.227 ± 2.621	47.096 ± 1.510	46.463 ± 0.592	0.331

CON=Control, BF=Basal Feed, CL=*Chaetomorpha linum*, CL1: 0.75% *Chaetomorpha linum* supplemented/kg BF, CL2: 1.5% *Chaetomorpha linum* supplemented/kg BF, CL3: 3% *Chaetomorpha linum* supplemented/kg BF. (Mean ± Standard error of mean)

## Discussion

CL is recognized as a macroalga with high abundance and has the potential to be utilized in daily life [31, 32]. Many studies have reported the bioactive potential of CL as a supplement or feed ingredient [10, 20, 32]. The use of marine products as supplements in animal feed provides an opportunity to increase animal growth performance because most marine products, such as microalgae and macroalgae, have suitable nutrient profiles [33, 34].

In the present study, the proximate test results of CL showed a protein content of 15.48%. This protein content significantly affected broilers’ growth performance after treatment with CL supplementation for 21 days. According to Saragih *et al*. [35], the green algae *Spirogyra jaoensis*, which has a protein content of 16%, has positive results in increasing the growth performance of broilers. It has also been reported that the incorporation of macroalgae *Ascophyllum nodosum*, whose protein content ranges from 11% to 16%, improved overall chicken growth and feed conversion [36]. Another study demonstrated an improvement in broiler growth performance following treatment with *Sargassum polycystum*, which contains 14.8% protein [37, 38]. Based on a previous study, protein content in feed is essential for broiler growth. The flavonoid test of CL revealed a flavonoid content of 0.16%. This content is sufficient as a broiler supplement. A previous study showed that 0.1% and 0.08% flavonoids supplemented the chicken diet, which enhanced chicken immunity [39]. Numerous studies have revealed a corresponding result that the administration of flavonoids in the range of 0.05%–0.2% improved chicken growth performance [40–42]. Flavonoids stimulate the immune barrier and induce antioxidant transfer, leading to the improvement of meat quality and chicken growth [41].

The study lasted for 21 days because it falls under the pre-starter (0–7 days), starter (8–14 days), and grower (14–21 days) phase [43–45]. In the poultry sector, these phases are important for understanding the dynamics of growth, and providing good nutrition from pre-starter to grower age will result in good broiler growth performance [46–48]. In addition, research on poultry nutrition during the finisher age is considered less effective because the growth curves tend to be constant [49].

The present study also found that administration of 1.5% and 3% CL gave positive results on weight gain and body weight for 21 days of treatment. According to Akinyemi and Adewole [50], there was an increase in the body weight of broilers after 2% brown seaweed was added. Choi *et al*. [51] and Kulshreshtha *et al*. [52] have also reported similar results in which 2% and 3% red seaweed positively affected chicken weight gain. Numerous algae and medicinal plants play a major role in biological reactions and metabolism [53]. FCR was also improved in the treatment group, which is similar to the result obtained by Mohammadigheisar *et al*. [54], in which a blend of red, green, and brown algae improved FCR and growth performance of broilers. There was a significant difference in feed intake of CL in the treatment groups compared to the CON group. This is in agreement with a study conducted by Balasubramanian *et al*. [55], who found that feeding red seaweed had a significant effect on the feed intake. Choi *et al*. [51] also found that the inclusion of fermented brown seaweed improves chicken feed intake. In contrast, several studies have reported no significant effect of algae inclusion on chicken diet [56–58]. We speculate that the discrepancy in results is due to the dosage and the palatability of species-specific algae used. The inclusion of algae in the chicken diet may reduce palatability and lead to reduced feed intake [59].

The small intestine is one of the prime organs in the digestive tract that absorbs nutrients [60]. Nutrient absorption efficiency depends on the small intestine’s structure, such as villi length, crypt depth, and the number of goblet cells that secrete mucin [61]. To achieve the optimal health of poultry, the small intestine should also be healthy. The results showed that the small intestine morphology had positive results after supplementation with CL in a broiler diet. In parallel, Saragih *et al*. [35] demonstrated that the inclusion of the green algae *S. jaoensis* improves the small intestine of broilers. The lengths of the duodenum, jejunum, and ileum villi showed a significant improvement in all treatments. Increased villi length enlarges the surface area of the small intestine and increases the efficiency of nutrient absorption through the growth of epithelial cells, mucosa and submucosa, and blood vessels, and frequently improves Lieberkeuhn gland growth [62, 63]. Several studies have shown that amino acids from poultry feed trigger protein synthesis during epithelial cell proliferation [64–66]. Moreover, protein content is responsible for epithelial cell regeneration and signal transduction in the small intestine [67], thus enhancing the efficiency of nutrient absorption [68]. In addition, flavonoid content plays a major role in antibacterial activity and stimulates epithelial cells to undergo mitosis. A previous study reported a similar result: the flavonoid from the *Phoenix dactylifera* activates the epithelial cells of intestinal villi to progress into the mitotic phase [29]. CL is rich in bioactive, flavonoids, fibers, and proteins [7]. These components improve the structure of small intestine.

Villi growth is also associated with crypt regeneration, and the acceleration of cell division also increases with increasing regeneration. The depth of the crypts indicates cell division and soon migrates upwards to become epithelial cells [69]. In the present study, the treatment group had the highest crypt depth observed. Animal feed proteins contribute to villi-crypt epithelial morphogenesis and the development of villi-crypt epithelial cells in the small intestine [70]. Flavonoids play a role in increasing villi height and crypt depth by protecting the small intestine walls from pathogens and reducing toxic compounds produced by microbes in epithelial cells [71–73]. It has also been reported that the growth of intestine villi is in parallel with the length of the villi-crypt; thus, a low ratio of crypt length contributes to efficient absorption [74]. CL proteins and flavonoids are responsible for villi-crypt regeneration.

The area and number of goblet cells in the treatment groups also showed the highest calculated number. The mucus secreted by goblet cells ensures the absorption of nutrients from the small intestine [75]. Goblet cells produce mucin, directly affecting intestinal microbial balance and facilitating nutrient transport [76]. The increase in goblet cell numbers in all treatment groups proves that CL is an effective antioxidant supplement for poultry.

Pectoral muscle (PM) growth is considered an important factor in evaluating the feeding regime and overall chicken growth [77, 78]. The reason is that PM has the lowest fat deposits compared to other parts [61, 79]. The present study showed that the administration of CL supplements at concentrations of 1.5% and 3% gave positive results on PM growth. The largest myofiber area was linear, with the largest fascicle area and the total myofiber count recorded. Myofiber area and size are determined by the growth rate and body weight of chicken [80]. Muscle growth is influenced by the protein content of broiler feed. Skeletal muscle growth in young birds appears primarily through the hypertrophy of pre-hatch myofibers [81]. The growth of pectoral muscle is due to the enlargement of muscle fibers and the results of satellite cells-mediated hypertrophy from these pre-existing fibers [72, 82–84]. Insulin-like growth factor-1 (IGF-1) production mediates the growth-promoting activities of growth hormone (GH) in poultry [85, 86]. IGF-1 and IGF-2 conduct skeletal myogenesis and contribute to the protein kinase B/the mammalian target of rapamycin pathway, which later regulates protein synthesis during hypertrophy [87]. GH, IGF-1, and IGF-2 activity is upregulated by adequate protein consumption in broilers [86]. Several studies have reported that the protein content of algae positively enhances protein synthesis and promotes muscle growth, eventually resulting in improved breast fillet yield in poultry [88–91]. The flavonoid in CL also possesses an antioxidant activity that enlarges the myofiber area and protects against free radicals [79, 92]. Other experiments have also yielded similar results in which flavonoids play a role in myofiber and fascicle growth [61, 72]. Several studies have also reported that flavonoid inclusion in chicken diets directly improves muscle weight [29, 93] and induces protein synthesis to promote muscle growth [94]. As the present study showed a significant effect of CL supplementation on PM weight, fascicle, myofiber area, and total number of myofiber per fascicle, it is clear that CL can be used as an alternative in poultry diet.

Supplements in poultry feed significantly affect meat quality. WHC [55, 95] is an indicator of meat quality. WHC refers to the capacity of cells to retain water and influences meat tenderness and juiciness [56, 96]. In the present study, treatment groups had a significantly higher WHC value than CON groups. This is in parallel with a study conducted by Balasubramanian *et al*. [55], who reported that administering red macroalgae *Halymenia palmata* to broilers improves WHC and reduces cooking loss. Another study by Qadri *et al*. [97] also corroborates that incorporating red seaweed *K. alvarezii* significantly increased WHC in broiler meat. Based on a previous study Hamzaoui *et al*. [98], the polysaccharide content of CL might play a major role in retaining the water content in the cell.

There were no significant differences in cholesterol content between the CON and treatment groups. On the other hand, the addition of *U. rigida* to the diet significantly reduced cholesterol content in broilers [14]. Although marine macroalgae are believed to have cholesterol-lowering effects [99, 100], another study corroborates the results of the present study, stating that rabbits fed with brown seaweed *Laminaria* spp. exhibit no significant cholesterol reduction [101]. The inclusion of *Macrocystis pyrifera* macroalgae in pig diet resulted in a higher cholesterol content [102]. Variation of algae diet results in species-specific and dose-dependent manner. Therefore, there is a need for further studies in different species and different doses.

## Conclusion

The inclusion of CL significantly improved the small intestine and pectoral muscle histomorphology, growth performance, and meat quality of broilers. This result proves that CL is a feasible supplement for animal feed, especially poultry, because of its rich nutrient content, such as crude protein and secondary metabolites.

## Authors’ Contributions

HTS: Conceptualized, analyzed the data, and wrote the manuscript. INF and DAS: Conducted the experiment and analyzed the data. ARC: Analyzed the data and drafted the manuscript. All authors have read, reviewed, and approved the final manuscript.
